# Efficacy of the Semicircular Inverted Internal Limiting Membrane Flap Technique for Macular Hole Retinal Detachment in Hyperopic Eyes: A Case Series

**DOI:** 10.7759/cureus.93149

**Published:** 2025-09-24

**Authors:** Masayuki Inuzuka, Masaomi Kubota, Norifumi Takaki, Toshio Hisatomi, Hirokazu Sakaguchi

**Affiliations:** 1 Department of Ophthalmology and Visual Sciences, Gifu University, Graduate School of Medical Sciences, Gifu, JPN; 2 Ophthalmology, Kubota Eye Clinic, Gifu, JPN; 3 Department of Ophthalmology, Graduate School of Medical Sciences, Kyushu University, Fukuoka, JPN; 4 Department of Ophthalmology and Visual Sciences, Graduate School of Biomedical Sciences, Hiroshima University, Hiroshima, JPN

**Keywords:** hyperopia, inverted internal limiting membrane flap, macular hole retinal detachment, optical coherence tomography, outer retinal layer restoration, pars plana vitrectomy, semicircular ilm flap technique, subretinal fluid absorption

## Abstract

We report anatomical and functional outcomes for three hyperopic phakic women with macular hole retinal detachment (MHRD) treated using a superiorly based semicircular inverted internal limiting membrane (ILM) flap. All patients underwent 25-gauge pars plana vitrectomy with Brilliant Blue G-assisted ILM manipulation, inversion of a superior semicircular flap to cover the macular hole, and 20% sulfur hexafluoride (SF₆) tamponade. Postoperative assessment included decimal best-corrected visual acuity (BCVA) and spectral-domain optical coherence tomography (OCT). All eyes achieved immediate macular hole closure, and complete retinal reattachment was attained after 18-24 months. Final BCVA improved in two eyes (from 0.1 to 0.4) and remained 0.2 in one eye, underscoring delayed but meaningful recovery in hyperopic MHRD treated with a semicircular inverted ILM flap. OCT demonstrated stepwise foveal restoration with variable ellipsoid zone/external limiting membrane (ELM/EZ) integrity across cases, and no intraoperative complications, macular hole reopening, or recurrent detachment were observed. These findings indicate that in hyperopic MHRD, a semicircular inverted ILM flap is feasible and mechanically reliable for early hole closure, while SRF absorption and functional recovery may require prolonged follow-up, likely reflecting the timeline of outer-retinal reconstruction.

## Introduction

Macular hole retinal detachment (MHRD) is an uncommon but vision-threatening condition in which a full-thickness macular hole precipitates neurosensory retinal detachment. Pars plana vitrectomy (PPV) with internal limiting membrane (ILM) peeling remains a commonly adopted surgical approach; to improve closure in refractory or large holes, the inverted ILM flap technique - folding a remnant ILM over the hole to serve as a scaffold for Müller cell-mediated glial bridging - has been widely utilized [1]. Meta-analyses suggest that, compared with conventional peeling, inverted flaps increase anatomical closure rates, while improvements in visual acuity may be similar at the study level [2]. Beyond highly myopic settings, successful use of inverted flaps has also been reported in macular holes coexistent with rhegmatogenous retinal detachment [3].

While MHRD predominantly occurs in highly myopic eyes, hyperopic eyes - with shorter axial lengths and thicker posterior poles - may exhibit slower retinal reattachment and prolonged subretinal fluid (SRF) retention, which can delay visual recovery. Treatment options include conventional ILM peeling and inverted ILM flap techniques; the latter may stabilize closure, yet does not guarantee rapid functional improvement. Persistent SRF following surgery for MH-associated retinal detachment has been documented, and macular hole closure can be achieved even over residual SRF [4, 5]. In parallel, the time course of outer-retinal reconstruction - particularly of the ellipsoid zone (EZ) and external limiting membrane (ELM) - has been linked to delayed yet meaningful functional recovery after successful closure [6, 7]. Regarding pathogenesis, the temporal relationship between posterior vitreous detachment and macular hole onset underscores the role of vitreomacular traction [8], while choroidal biomechanical factors may influence macular contour and fluid dynamics [9].

Here, we report three cases of three hyperopic phakic women with MHRD who underwent repair using a superiorly based semicircular inverted ILM flap. We evaluate anatomical and functional outcomes over extended follow-up, emphasizing (i) the sequence from early hole closure to delayed retinal reattachment, (ii) the temporal relationship between SRF absorption and EZ/ELM restoration, and (iii) the surgical rationale, including conservative management of subfoveal SRF, to inform procedure selection, postoperative counseling, and follow-up strategies in hyperopic MHRD.

## Case presentation

Overview

Three hyperopic phakic women with MHRD were managed using a standardized, superiorly based semicircular inverted ILM flap. At presentation, spherical equivalents were +6.00, +2.75, and +1.25 diopters (D), and axial lengths were 20.87, 21.94, and 22.39 mm, respectively. Baseline best-corrected visual acuities (BCVA) were 0.2, 0.1, and 0.1. Baseline demographics and comorbidities/medications are summarized in Table 1.

Patients and Study Design

This case series included three hyperopic phakic women with MHRD treated at Gifu University Hospital. Hyperopia was defined as a spherical equivalent (SE) greater than +0.50 diopters. The study adhered to the tenets of the Declaration of Helsinki, and written informed consent for both treatment and publication was obtained from all patients.

Surgical Procedure

Following phacoemulsification and intraocular lens implantation, 25-gauge PPV was performed. After detachment of the posterior hyaloid, the ILM was stained with Brilliant Blue G. The inferior ILM was removed as completely as possible; when residual ILM fragments were present at the hole margin, they were gently inserted into the macular hole. A superiorly based semicircular ILM flap was preserved, inverted to cover the hole, and stabilized with a small amount of viscoelastic. Fluid-air exchange was then performed, followed by gas tamponade with 20% sulfur hexafluoride (SF₆). Patients maintained a prone position for several days.

Surgical Rationale

Subfoveal SRF was not aspirated to minimize foveal trauma and iatrogenic hole enlargement, preserve flap stability, and allow retinal pigment epithelium (RPE) pump-driven reabsorption.

Postoperative Assessment

Follow-up included decimal BCVA (Landolt C charts) and spectral-domain OCT at standardized intervals (e.g., week 1; months 1, 3, 6, 12, 18, and 24, as available). Primary outcomes were anatomical MH closure and retinal reattachment. Secondary outcomes were changes in BCVA and integrity of outer-retinal layers (EZ/ELM).

Case 1

**Figure 1:**
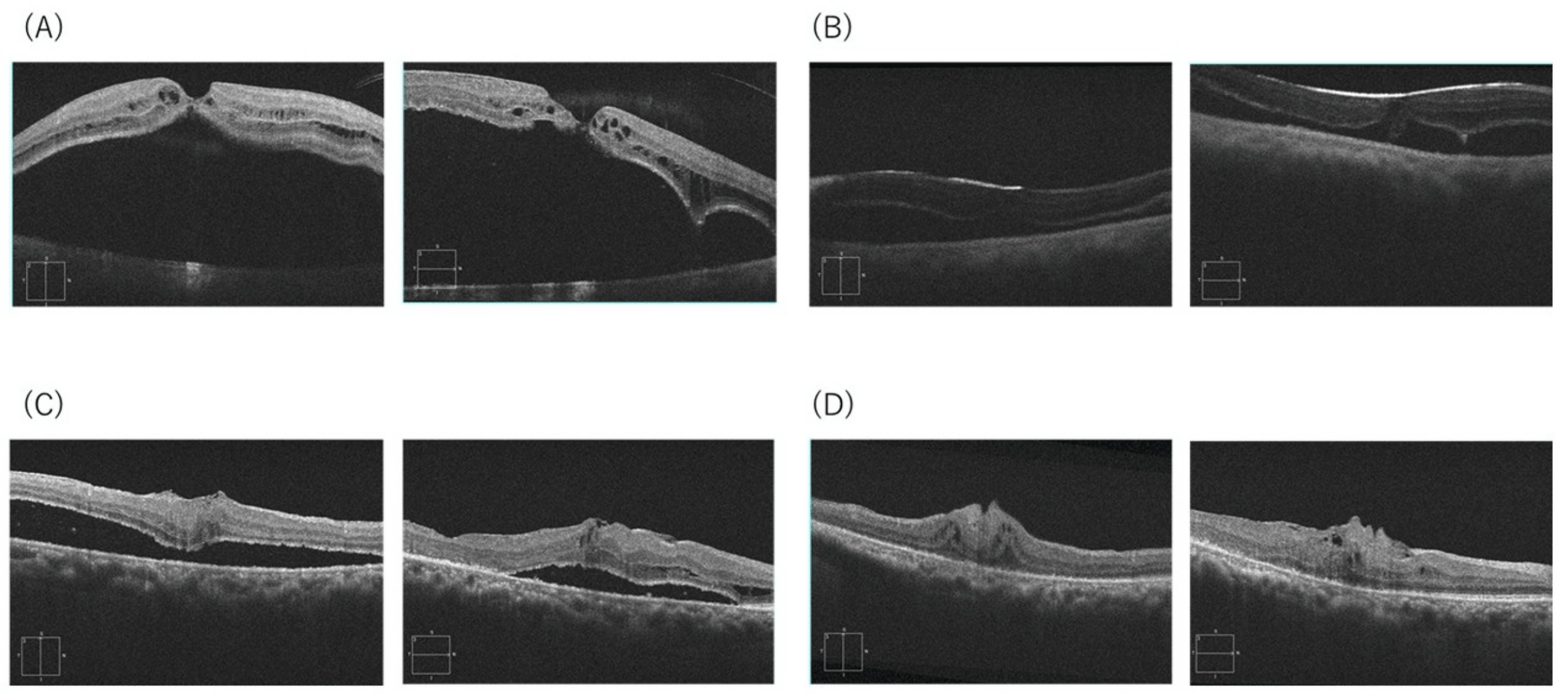
OCT images of Case 1 (A) Preoperative MHRD. (B) Post-op Week 1: inverted ILM flap covering the MH with early edge separation. (C) Month 6: progressive approximation of MH edges and decreased SRF. (D) Month 18: complete SRF absorption with restored foveal contour. SE +6.00 D; AL 20.87 mm; final BCVA 0.2. OCT: optical coherence tomography; MHRD: macular hole retinal detachment; ILM: internal limiting membrane; MH: macular hole; SRF: subretinal fluid; SE: spherical equivalent; AL: axial length; BCVA: best-corrected visual acuity

A hyperopic phakic woman presented with central visual disturbance and MHRD (SE +6.00 D; axial length 20.87 mm; baseline BCVA 0.2). The standardized procedure achieved complete flap coverage of the hole. Early postoperative OCT showed edge separation that progressively diminished. SRF resolved at 18 months. Final BCVA was 0.2. No intraoperative complications, macular hole reopening, or recurrent retinal detachment occurred (Figure 1).

Case 2

**Figure 2:**
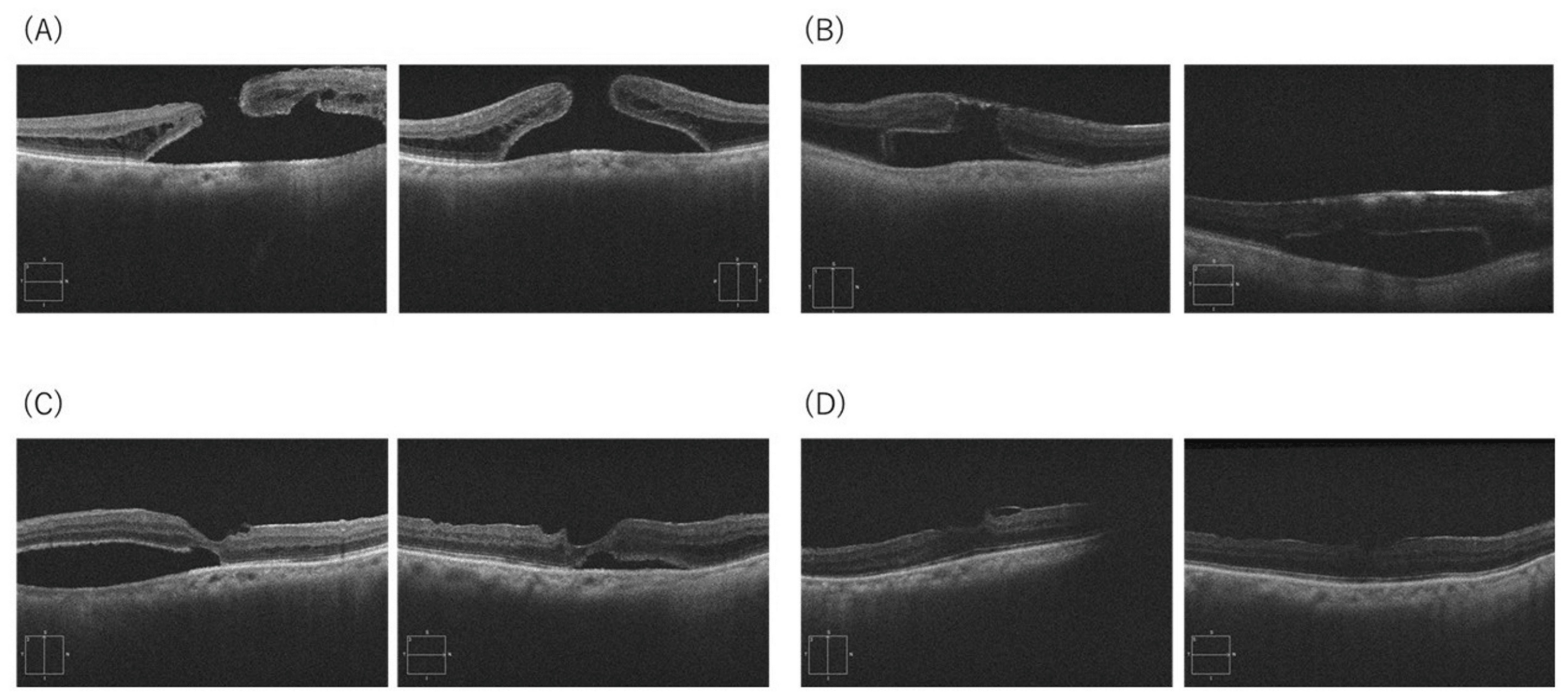
OCT images of Case 2 (A) Preoperative MHRD. (B) Post-op Week 1: stable flap coverage with edge separation. (C) Month 3: progressive edge approximation and SRF decrease. (D) Month 24: complete SRF absorption and sustained MH closure. SE +2.75 D; AL 21.94 mm; BCVA improved from 0.1 to 0.4. OCT: optical coherence tomography; MHRD: macular hole retinal detachment; SRF: subretinal fluid; MH: macular hole; SE: spherical equivalent; AL: axial length; BCVA: best-corrected visual acuity

A hyperopic phakic woman presented with central visual disturbance and MHRD (SE +2.75 D; axial length 21.94 mm; baseline BCVA 0.1). OCT demonstrated a well-positioned inverted flap with gradual approximation of the MH edges and decreasing SRF. Complete SRF absorption was documented at 24 months. Final BCVA improved from 0.1 to 0.4. No intraoperative complications, MH reopening, or recurrent detachment were observed (Figure 2).

Case 3

**Figure 3:**
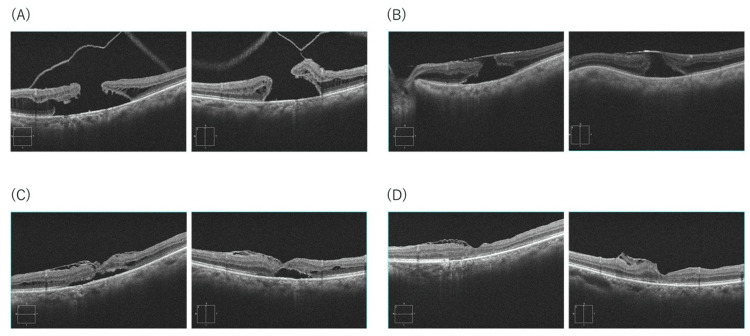
OCT images of Case 3 (A) Preoperative MHRD. (B) Post-op Week 1: prompt flap coverage of the MH. (C) Month 3: continued reattachment with stepwise foveal restoration. (D) Month 18: complete SRF absorption and stable closure. SE +1.25 D; AL 22.39 mm; BCVA improved from 0.1 to 0.4. OCT: optical coherence tomography; MHRD: macular hole retinal detachment; MH: macular hole; SRF: subretinal fluid; SE: spherical equivalent; AL: axial length; BCVA: best-corrected visual acuity

A hyperopic phakic woman presented with central visual disturbance and MHRD (SE +1.25 D; axial length 22.39 mm; baseline BCVA 0.1). OCT showed prompt flap coverage with sustained closure and progressive foveal reconstitution. SRF resolved by 18 months. Final BCVA improved from 0.1 to 0.4. No intraoperative complications, MH reopening, or recurrent detachment occurred (Figure 3).

Note: Exact postoperative time points for the representative OCT images are specified in the figure captions (e.g., Post-op Week 1, Month 3, Month 18/24).

Table 1 shows the clinical outcomes of all three cases.

**Table 1:** Clinical Characteristics and Outcomes All cases underwent phacoemulsification with intraocular lens implantation followed by 25-gauge pars plana vitrectomy and a superiorly based semicircular inverted ILM flap; see the case presentation for details. SE: spherical equivalent; BCVA: best-corrected visual acuity; SRF: subretinal fluid; MH: macular hole

Case	Age / Sex	Axial Length (mm)	Comorbidities / Past History	Preoperative SE (D)	Preoperative BCVA	Final BCVA	MH Closure	SRF Resolution (month)	Complications / Recurrence
①	81 / F	20.87	None	+6.00	0.2	0.2	Yes	18	None
②	72 / F	21.94	Diabetes mellitus, Hypertension	+2.75	0.1	0.4	Yes	24	None
③	85 / F	22.39	Hypertension	+1.25	0.1	0.4	Yes	18	None

## Discussion

This case series provides insight into the structural and functional trajectory of hyperopic MHRD treated with a semicircular inverted ILM flap. All three eyes achieved early anatomical macular hole closure with stable flap positioning and no intraoperative complications, reopening, or recurrent detachment. However, complete retinal reattachment was delayed, with SRF absorbing over 18, 24, and 18 months, and visual recovery lagging behind anatomic success. This dissociation underscores that, particularly in hyperopic MHRD, functional improvement may require many months beyond early closure, likely reflecting the time course of EZ/ELM reconstruction [6,7].

Our findings align with prior reports showing that inverted flaps promote reliable anatomical closure in challenging macular holes, while improvements in visual acuity may be comparable to conventional ILM peeling at the study level [2]. In highly myopic cohorts with MHRD, randomized or comparative data suggest that inverted ILM insertion/flap produces high closure rates and favorable foveal contour recovery relative to standard peeling [10,11]. Importantly, persistent SRF after MHRD repair is well recognized and can coexist with successful hole closure [4,5]. In our hyperopic series, SRF clearance required 18-24 months, toward the longer end of published ranges. Given small samples and anatomical differences, we do not claim a systematic kinetic difference from high-myopia studies; we observed no qualitative difference in the closure sequence beyond the prolonged SRF course.

Beyond providing a mechanical scaffold, the inverted ILM flap plausibly acts as a biological matrix that stimulates Müller cell-mediated glial bridging [1]. Perimacular proliferative changes following inverted-flap surgery can occur, and a planned semicircular approach may facilitate a monolayer ILM that completely covers the hole, conceptually reducing the need for multilayer stacking, and thereby supporting EZ/ELM reconstruction, which has been linked to delayed yet meaningful visual recovery [6,7]. In our cohort, limiting the extent of ILM dissection appeared to reduce intraoperative manipulation and yielded stable flap positioning throughout follow-up, supporting the mechanical reliability of the semicircular technique and its consistency with prior observations in challenging macular holes [1].

Several mechanisms may explain the prolonged SRF retention in hyperopic MHRD. Compared with highly myopic eyes - where axial elongation and scleral thinning may facilitate retinal flattening - the shorter axial length and thicker posterior pole in hyperopia could offer greater mechanical resistance to rapid reattachment, with additional contributions from choroidal fluid dynamics [9]. Moreover, the temporal relationship between posterior vitreous detachment and macular hole onset supports a tractional component that may promote centripetal shortening and glial remodeling [8]. Finally, the semicircular flap likely created an effective seal over the hole; combined with our conservative approach of not aspirating subfoveal SRF, egress through the hole may have been limited, shifting reliance to RPE pump-driven clearance. Taken together, these anatomic and procedural factors plausibly account for the extended SRF timeline observed here.

Clinical implications follow from these observations. First, surgeons should counsel patients that in hyperopic MHRD, anatomical closure does not guarantee prompt visual recovery; improvement often depends on EZ/ELM restoration over many months [6,7]. Second, extended follow-up is appropriate to monitor outer-retinal remodeling and confirm durable reattachment. Third, flap design (semicircular vs circular) and SRF management might be individualized, balancing flap stability against the potential benefits of targeted SRF evacuation in select cases. Typical risks after MHRD repair include macular hole reopening, recurrent retinal detachment, and flap displacement; none occurred in our cohort, although the small sample precludes firm conclusions about safety. Alternative strategies such as autologous ILM transplantation may be considered for refractory holes on a case-by-case basis [12].

This study has limitations. It is a small, retrospective series without a control group, limiting generalizability and causal inference; imaging intervals and follow-up durations varied modestly. Formal assessment of metamorphopsia (e.g., M-CHARTS) was not performed and should be incorporated in future work together with patient-reported outcomes. Larger, comparative studies are needed to define hyperopia-specific healing kinetics, the impact of flap geometry, and the role of targeted SRF drainage on the pace of outer-retinal reconstruction and vision.

In summary, the semicircular inverted ILM flap was feasible and anatomically reliable for hyperopic MHRD, achieving early macular hole closure without recurrence. Nevertheless, SRF absorption and visual recovery followed a prolonged course, largely governed by outer-retinal restoration. Surgical planning should therefore address not only hole closure but also strategies that may influence SRF clearance and functional outcomes, with realistic postoperative counseling and extended follow-up.

## Conclusions

The superiorly based semicircular inverted ILM flap was feasible in hyperopic MHRD, yielding early macular hole closure with stable flap positioning and no intraoperative complications, reopening, or recurrent detachment. However, complete retinal reattachment and visual recovery followed a delayed trajectory: SRF resolved at 18-24 months, and final BCVA improved to 0.4 in two eyes while remaining 0.2 in one. These findings emphasize that in hyperopic eyes, anatomical success may precede functional recovery by many months, likely reflecting the timeline of EZ/ELM restoration. Surgical planning and counseling should therefore consider not only hole closure but also SRF management and the expectation of prolonged follow-up.
